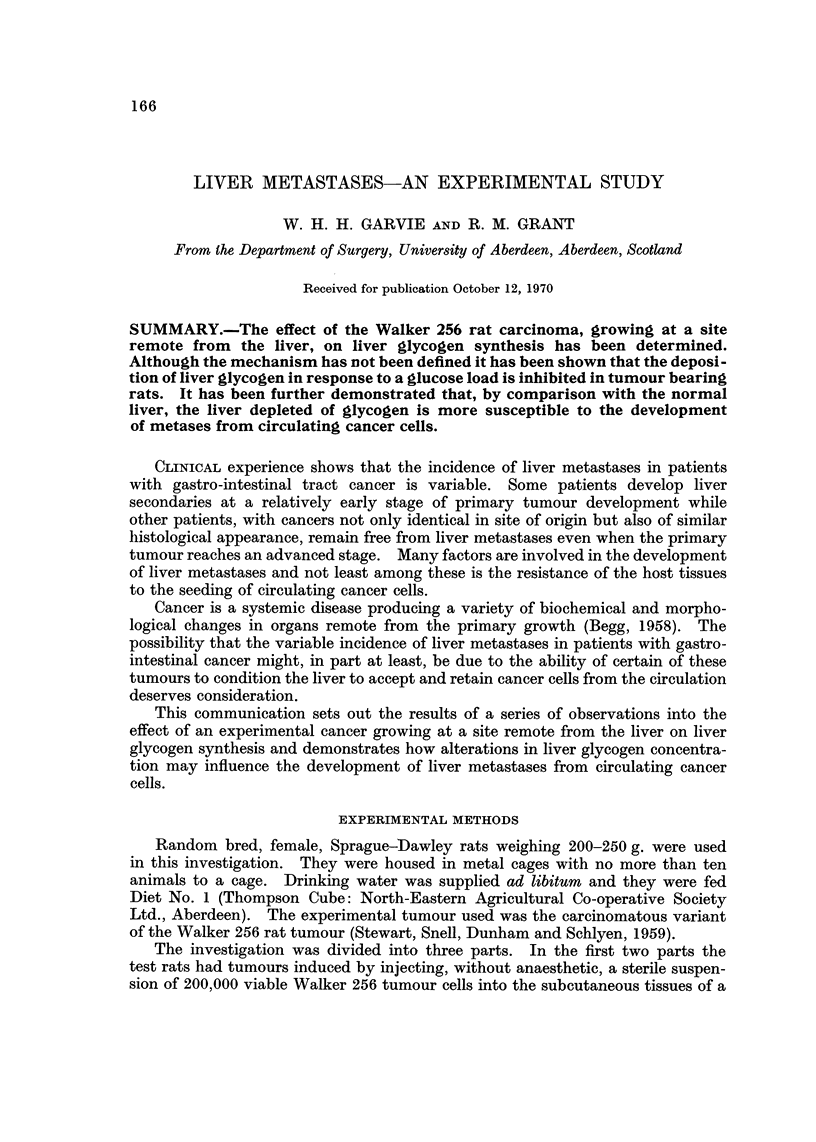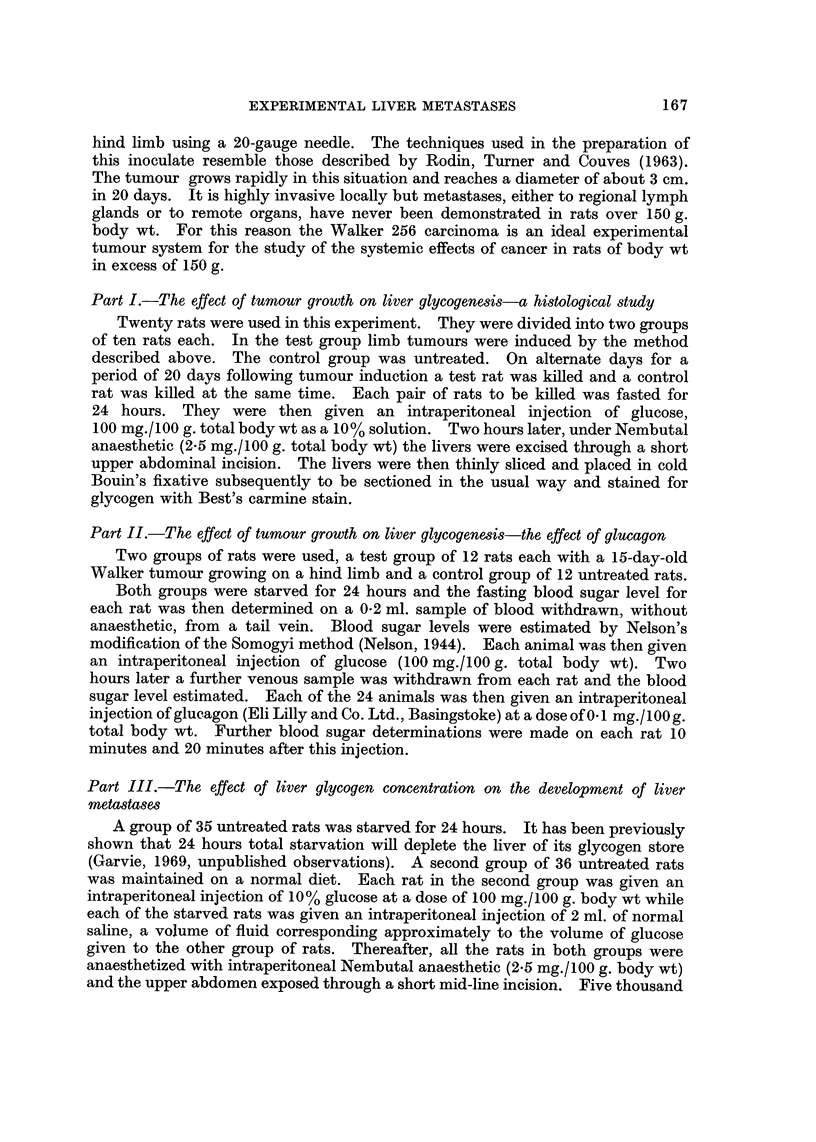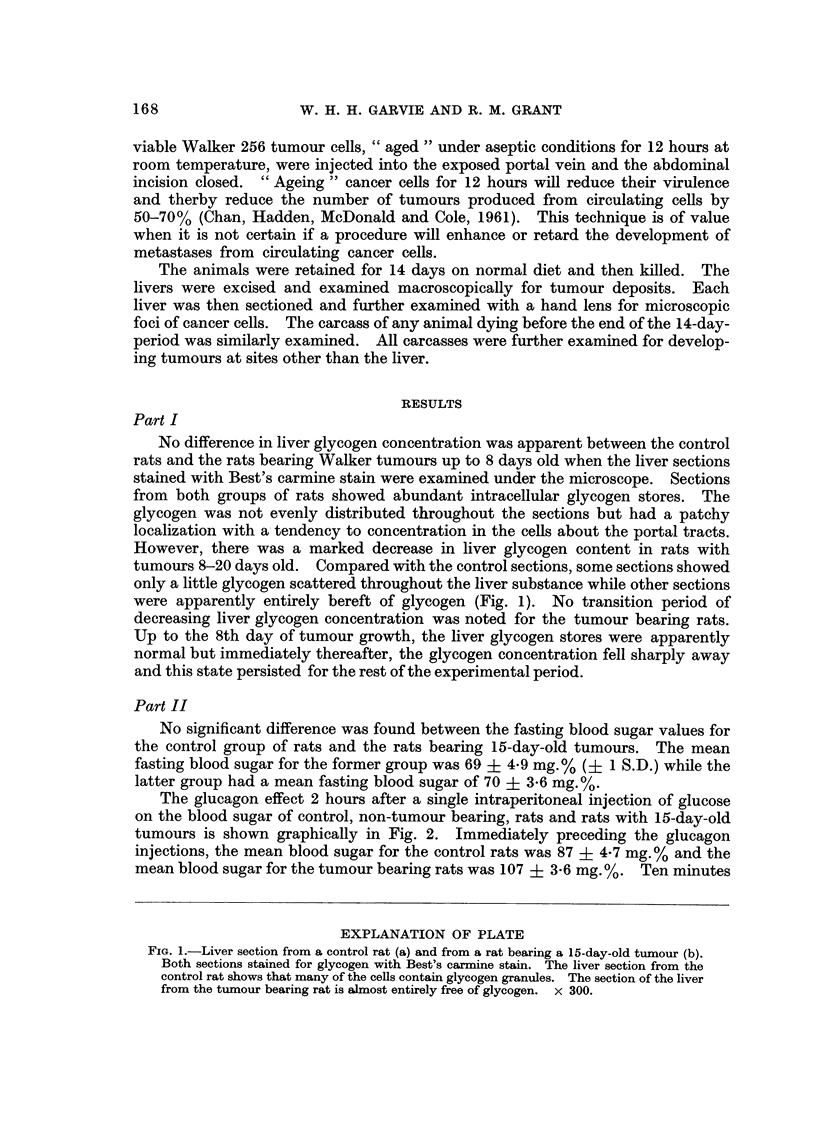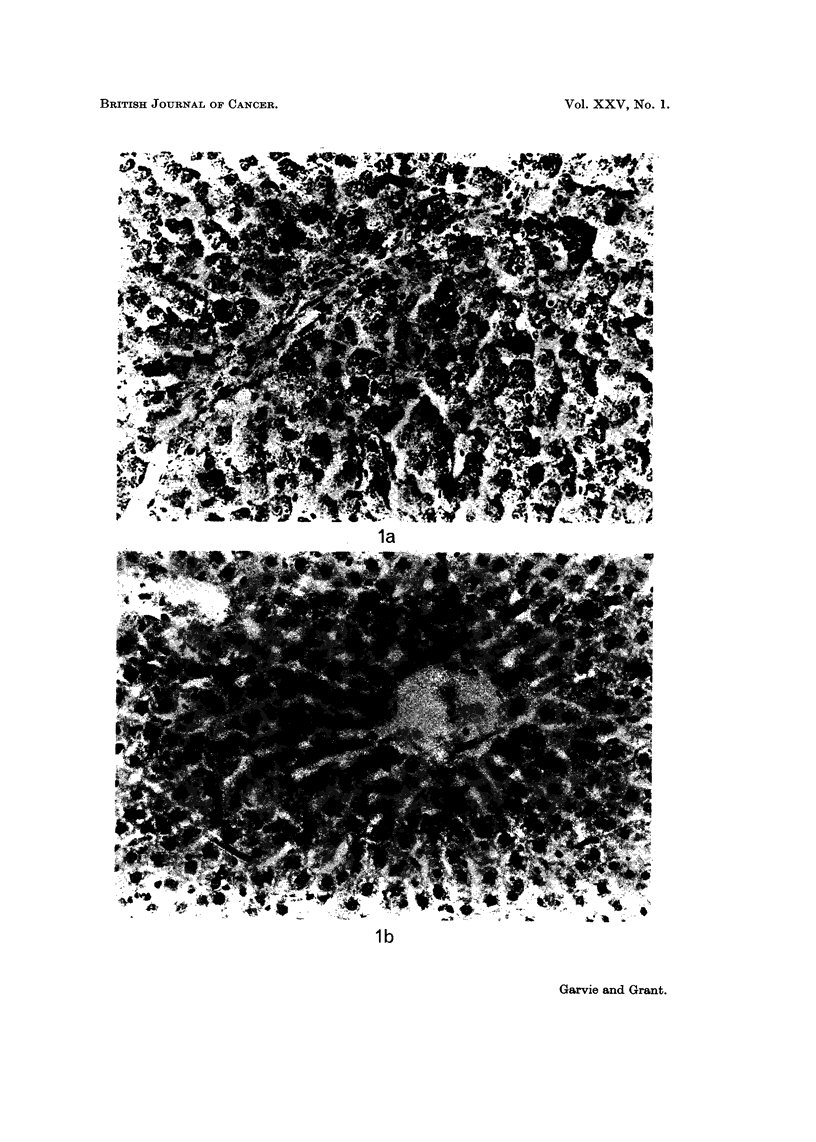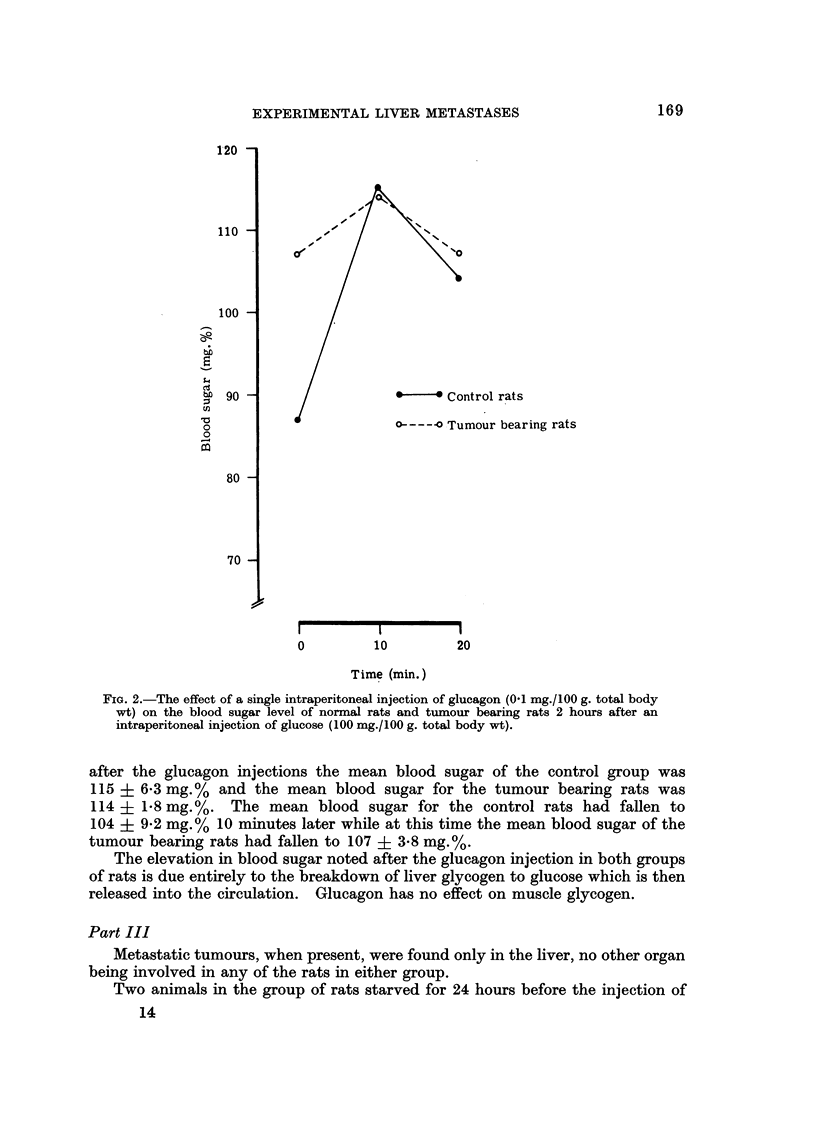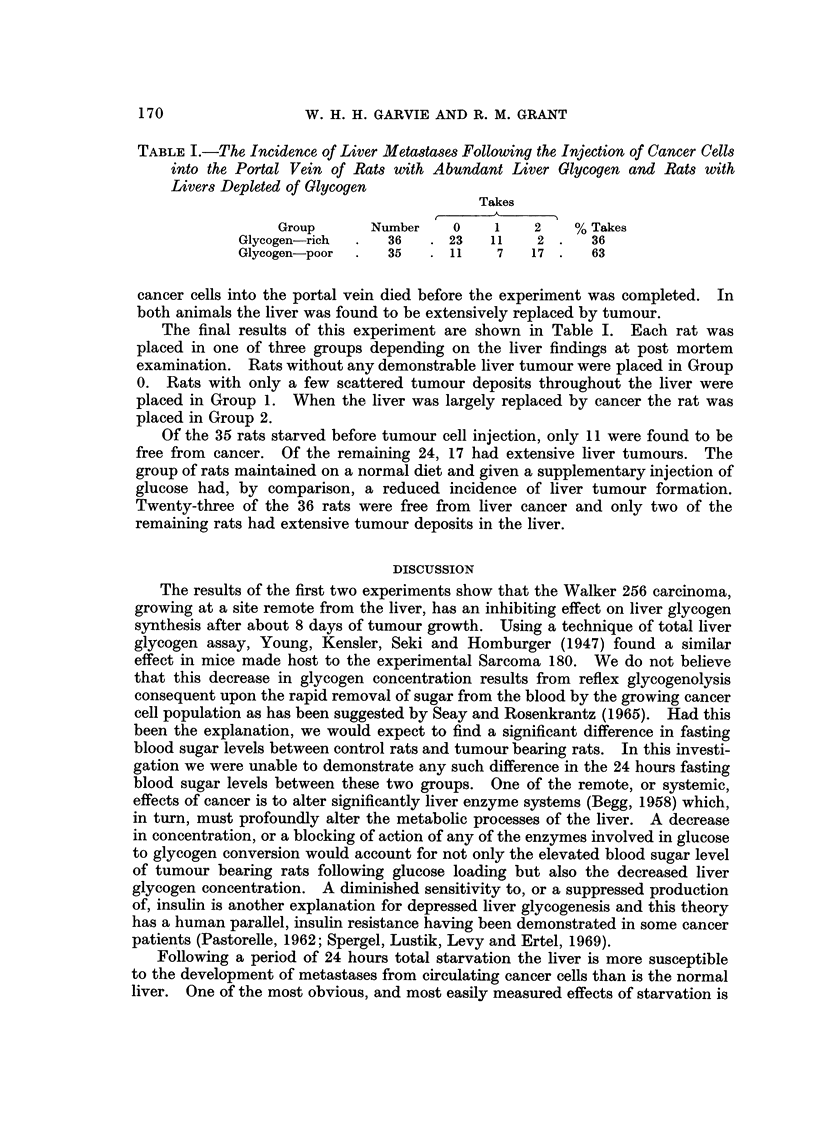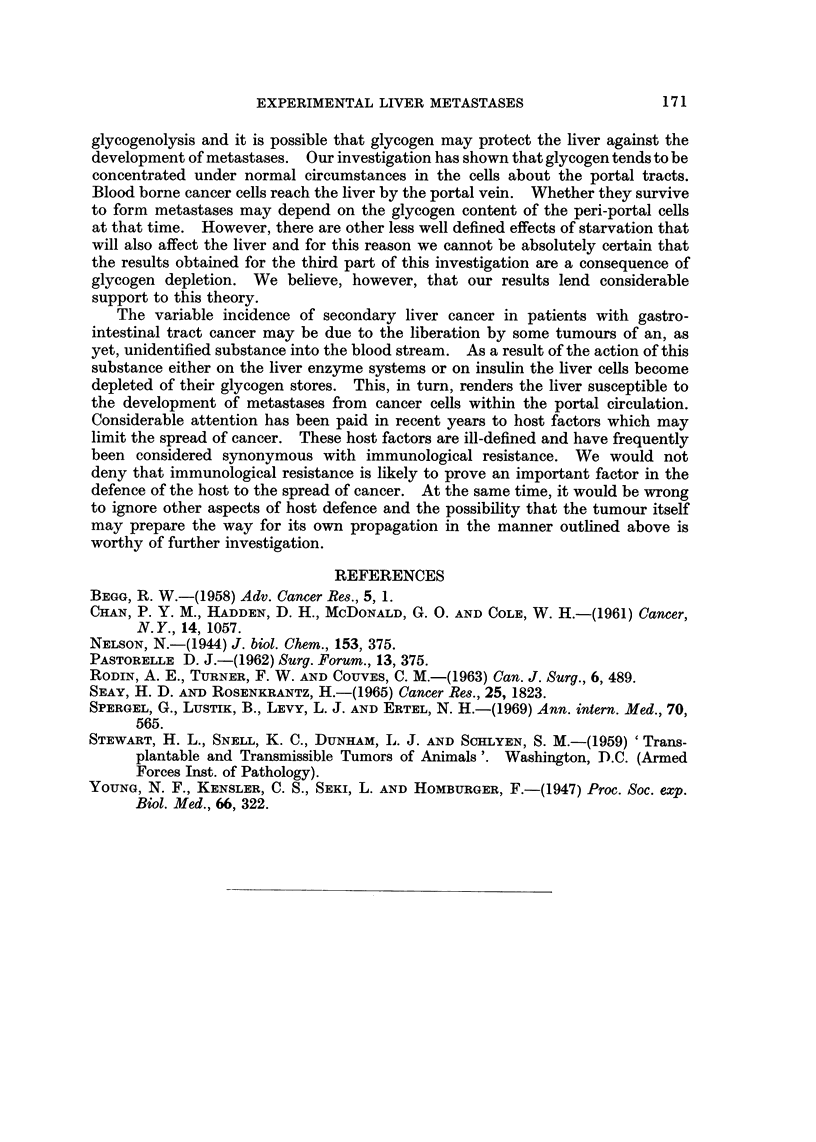# Liver Metastases—an Experimental Study

**DOI:** 10.1038/bjc.1971.23

**Published:** 1971-03

**Authors:** W. H. H. Garvie, R. M. Grant

## Abstract

**Images:**


### LIVER METASTASES—AN EXPERIMENTAL STUDY

W. H. H. GARVIE AND R. M. GRANT

*From the Department of Surgery, University of Aberdeen, Aberdeen, Scotland*

Received for publication October 12, 1970

**SUMMARY.—The effect of the Walker 256 rat carcinoma, growing at a site remote from the liver, on liver glycogen synthesis has been determined. Although the mechanism has not been defined it has been shown that the deposition of liver glycogen in response to a glucose load is inhibited in tumour bearing rats. It has been further demonstrated that, by comparison with the normal liver, the liver depleted of glycogen is more susceptible to the development of metases from circulating cancer cells.**

CLINICAL experience shows that the incidence of liver metastases in patients with gastro-intestinal tract cancer is variable. Some patients develop liver secondaries at a relatively early stage of primary tumour development while other patients, with cancers not only identical in site of origin but also of similar histological appearance, remain free from liver metastases even when the primary tumour reaches an advanced stage. Many factors are involved in the development of liver metastases and not least among these is the resistance of the host tissues to the seeding of circulating cancer cells.

Cancer is a systemic disease producing a variety of biochemical and morphological changes in organs remote from the primary growth (Begg, 1958). The possibility that the variable incidence of liver metastases in patients with gastro-intestinal cancer might, in part at least, be due to the ability of certain of these tumours to condition the liver to accept and retain cancer cells from the circulation deserves consideration.

This communication sets out the results of a series of observations into the effect of an experimental cancer growing at a site remote from the liver on liver glycogen synthesis and demonstrates how alterations in liver glycogen concentration may influence the development of liver metastases from circulating cancer cells.

### EXPERIMENTAL METHODS

Random bred, female, Sprague–Dawley rats weighing 200–250 g. were used in this investigation. They were housed in metal cages with no more than ten animals to a cage. Drinking water was supplied *ad libitum* and they were fed Diet No. 1 (Thompson Cube: North-Eastern Agricultural Co-operative Society Ltd., Aberdeen). The experimental tumour used was the carcinomatous variant of the Walker 256 rat tumour (Stewart, Snell, Dunham and Schlyen, 1959).

The investigation was divided into three parts. In the first two parts the test rats had tumours induced by injecting, without anaesthetic, a sterile suspension of 200,000 viable Walker 256 tumour cells into the subcutaneous tissues of a hind limb using a 20-gauge needle. The techniques used in the preparation of this inoculate resemble those described by Rodin, Turner and Couves (1963). The tumour grows rapidly in this situation and reaches a diameter of about 3 cm. in 20 days. It is highly invasive locally but metastases, either to regional lymph glands or to remote organs, have never been demonstrated in rats over 150 g. body wt. For this reason the Walker 256 carcinoma is an ideal experimental tumour system for the study of the systemic effects of cancer in rats of body wt in excess of 150 g.

*Part I.—The effect of tumour growth on liver glycogenesis—a histological study*

Twenty rats were used in this experiment. They were divided into two groups of ten rats each. In the test group limb tumours were induced by the method described above. The control group was untreated. On alternate days for a period of 20 days following tumour induction a test rat was killed and a control rat was killed at the same time. Each pair of rats to be killed was fasted for 24 hours. They were then given an intraperitoneal injection of glucose, 100 mg./100 g. total body wt as a 10% solution. Two hours later, under Nembutal anaesthetic (2·5 mg./100 g. total body wt) the livers were excised through a short upper abdominal incision. The livers were then thinly sliced and placed in cold Bouin's fixative subsequently to be sectioned in the usual way and stained for glycogen with Best's carmine stain.

*Part II.—The effect of tumour growth on liver glycogenesis—the effect of glucagon*

Two groups of rats were used, a test group of 12 rats each with a 15-day-old Walker tumour growing on a hind limb and a control group of 12 untreated rats.

Both groups were starved for 24 hours and the fasting blood sugar level for each rat was then determined on a 0·2 ml. sample of blood withdrawn, without anaesthetic, from a tail vein. Blood sugar levels were estimated by Nelson's modification of the Somogyi method (Nelson, 1944). Each animal was then given an intraperitoneal injection of glucose (100 mg./100 g. total body wt). Two hours later a further venous sample was withdrawn from each rat and the blood sugar level estimated. Each of the 24 animals was then given an intraperitoneal injection of glucagon (Eli Lilly and Co. Ltd., Basingstoke) at a dose of 0·1 mg./100 g. total body wt. Further blood sugar determinations were made on each rat 10 minutes and 20 minutes after this injection.

*Part III.—The effect of liver glycogen concentration on the development of liver metastases*

A group of 35 untreated rats was starved for 24 hours. It has been previously shown that 24 hours total starvation will deplete the liver of its glycogen store (Garvie, 1969, unpublished observations). A second group of 36 untreated rats was maintained on a normal diet. Each rat in the second group was given an intraperitoneal injection of 10% glucose at a dose of 100 mg./100 g. body wt while each of the starved rats was given an intraperitoneal injection of 2 ml. of normal saline, a volume of fluid corresponding approximately to the volume of glucose given to the other group of rats. Thereafter, all the rats in both groups were anaesthetized with intraperitoneal Nembutal anaesthetic (2·5 mg./100 g. body wt) and the upper abdomen exposed through a short mid-line incision. Five thousand viable Walker 256 tumour cells, " aged " under aseptic conditions for 12 hours at room temperature, were injected into the exposed portal vein and the abdominal incision closed. " Ageing " cancer cells for 12 hours will reduce their virulence and therby reduce the number of tumours produced from circulating cells by 50–70% (Chan, Hadden, McDonald and Cole, 1961). This technique is of value when it is not certain if a procedure will enhance or retard the development of metastases from circulating cancer cells.

The animals were retained for 14 days on normal diet and then killed. The livers were excised and examined macroscopically for tumour deposits. Each liver was then sectioned and further examined with a hand lens for microscopic foci of cancer cells. The carcass of any animal dying before the end of the 14-day-period was similarly examined. All carcasses were further examined for developing tumours at sites other than the liver.

### RESULTS

*Part I*

No difference in liver glycogen concentration was apparent between the control rats and the rats bearing Walker tumours up to 8 days old when the liver sections stained with Best's carmine stain were examined under the microscope. Sections from both groups of rats showed abundant intracellular glycogen stores. The glycogen was not evenly distributed throughout the sections but had a patchy localization with a tendency to concentration in the cells about the portal tracts. However, there was a marked decrease in liver glycogen content in rats with tumours 8–20 days old. Compared with the control sections, some sections showed only a little glycogen scattered throughout the liver substance while other sections were apparently entirely bereft of glycogen (Fig. 1). No transition period of decreasing liver glycogen concentration was noted for the tumour bearing rats. Up to the 8th day of tumour growth, the liver glycogen stores were apparently normal but immediately thereafter, the glycogen concentration fell sharply away and this state persisted for the rest of the experimental period.

*Part II*

No significant difference was found between the fasting blood sugar values for the control group of rats and the rats bearing 15-day-old tumours. The mean fasting blood sugar for the former group was 69 $\pm$ 4·9 mg.% ($\pm$ 1 S.D.) while the latter group had a mean fasting blood sugar of 70 $\pm$ 3·6 mg.%.

The glucagon effect 2 hours after a single intraperitoneal injection of glucose on the blood sugar of control, non-tumour bearing, rats and rats with 15-day-old tumours is shown graphically in Fig. 2. Immediately preceding the glucagon injections, the mean blood sugar for the control rats was 87 $\pm$ 4·7 mg.% and the mean blood sugar for the tumour bearing rats was 107 $\pm$ 3·6 mg.%. Ten minutes

---

EXPLANATION OF PLATE

FIG. 1.—Liver section from a control rat (a) and from a rat bearing a 15-day-old tumour (b). Both sections stained for glycogen with Best's carmine stain. The liver section from the control rat shows that many of the cells contain glycogen granules. The section of the liver from the tumour bearing rat is almost entirely free of glycogen. $\times$ 300.

1a

1b

Garvie and Grant.

FIG. 2.—The effect of a single intraperitoneal injection of glucagon (0·1 mg./100 g. total body wt) on the blood sugar level of normal rats and tumour bearing rats 2 hours after an intraperitoneal injection of glucose (100 mg./100 g. total body wt).

after the glucagon injections the mean blood sugar of the control group was $115 \pm 6{\cdot}3$ mg.% and the mean blood sugar for the tumour bearing rats was $114 \pm 1{\cdot}8$ mg.%. The mean blood sugar for the control rats had fallen to $104 \pm 9{\cdot}2$ mg.% 10 minutes later while at this time the mean blood sugar of the tumour bearing rats had fallen to $107 \pm 3{\cdot}8$ mg.%.

The elevation in blood sugar noted after the glucagon injection in both groups of rats is due entirely to the breakdown of liver glycogen to glucose which is then released into the circulation. Glucagon has no effect on muscle glycogen.

*Part III*

Metastatic tumours, when present, were found only in the liver, no other organ being involved in any of the rats in either group.

Two animals in the group of rats starved for 24 hours before the injection of

TABLE I.—*The Incidence of Liver Metastases Following the Injection of Cancer Cells into the Portal Vein of Rats with Abundant Liver Glycogen and Rats with Livers Depleted of Glycogen*

| Group | Number | Takes 0 | 1 | 2 | % Takes |
|---|---|---|---|---|---|
| Glycogen—rich | 36 | 23 | 11 | 2 | 36 |
| Glycogen—poor | 35 | 11 | 7 | 17 | 63 |

cancer cells into the portal vein died before the experiment was completed. In both animals the liver was found to be extensively replaced by tumour.

The final results of this experiment are shown in Table I. Each rat was placed in one of three groups depending on the liver findings at post mortem examination. Rats without any demonstrable liver tumour were placed in Group 0. Rats with only a few scattered tumour deposits throughout the liver were placed in Group 1. When the liver was largely replaced by cancer the rat was placed in Group 2.

Of the 35 rats starved before tumour cell injection, only 11 were found to be free from cancer. Of the remaining 24, 17 had extensive liver tumours. The group of rats maintained on a normal diet and given a supplementary injection of glucose had, by comparison, a reduced incidence of liver tumour formation. Twenty-three of the 36 rats were free from liver cancer and only two of the remaining rats had extensive tumour deposits in the liver.

### DISCUSSION

The results of the first two experiments show that the Walker 256 carcinoma, growing at a site remote from the liver, has an inhibiting effect on liver glycogen synthesis after about 8 days of tumour growth. Using a technique of total liver glycogen assay, Young, Kensler, Seki and Homburger (1947) found a similar effect in mice made host to the experimental Sarcoma 180. We do not believe that this decrease in glycogen concentration results from reflex glycogenolysis consequent upon the rapid removal of sugar from the blood by the growing cancer cell population as has been suggested by Seay and Rosenkrantz (1965). Had this been the explanation, we would expect to find a significant difference in fasting blood sugar levels between control rats and tumour bearing rats. In this investigation we were unable to demonstrate any such difference in the 24 hours fasting blood sugar levels between these two groups. One of the remote, or systemic, effects of cancer is to alter significantly liver enzyme systems (Begg, 1958) which, in turn, must profoundly alter the metabolic processes of the liver. A decrease in concentration, or a blocking of action of any of the enzymes involved in glucose to glycogen conversion would account for not only the elevated blood sugar level of tumour bearing rats following glucose loading but also the decreased liver glycogen concentration. A diminished sensitivity to, or a suppressed production of, insulin is another explanation for depressed liver glycogenesis and this theory has a human parallel, insulin resistance having been demonstrated in some cancer patients (Pastorelle, 1962; Spergel, Lustik, Levy and Ertel, 1969).

Following a period of 24 hours total starvation the liver is more susceptible to the development of metastases from circulating cancer cells than is the normal liver. One of the most obvious, and most easily measured effects of starvation is glycogenolysis and it is possible that glycogen may protect the liver against the development of metastases. Our investigation has shown that glycogen tends to be concentrated under normal circumstances in the cells about the portal tracts. Blood borne cancer cells reach the liver by the portal vein. Whether they survive to form metastases may depend on the glycogen content of the peri-portal cells at that time. However, there are other less well defined effects of starvation that will also affect the liver and for this reason we cannot be absolutely certain that the results obtained for the third part of this investigation are a consequence of glycogen depletion. We believe, however, that our results lend considerable support to this theory.

The variable incidence of secondary liver cancer in patients with gastro-intestinal tract cancer may be due to the liberation by some tumours of an, as yet, unidentified substance into the blood stream. As a result of the action of this substance either on the liver enzyme systems or on insulin the liver cells become depleted of their glycogen stores. This, in turn, renders the liver susceptible to the development of metastases from cancer cells within the portal circulation. Considerable attention has been paid in recent years to host factors which may limit the spread of cancer. These host factors are ill-defined and have frequently been considered synonymous with immunological resistance. We would not deny that immunological resistance is likely to prove an important factor in the defence of the host to the spread of cancer. At the same time, it would be wrong to ignore other aspects of host defence and the possibility that the tumour itself may prepare the way for its own propagation in the manner outlined above is worthy of further investigation.

### REFERENCES

Begg, R. W.—(1958) *Adv. Cancer Res.*, **5**, 1.

Chan, P. Y. M., Hadden, D. H., McDonald, G. O. and Cole, W. H.—(1961) *Cancer, N.Y.*, **14**, 1057.

Nelson, N.—(1944) *J. biol. Chem.*, **153**, 375.

Pastorelle D. J.—(1962) *Surg. Forum.*, **13**, 375.

Rodin, A. E., Turner, F. W. and Couves, C. M.—(1963) *Can. J. Surg.*, **6**, 489.

Seay, H. D. and Rosenkrantz, H.—(1965) *Cancer Res.*, **25**, 1823.

Spergel, G., Lustik, B., Levy, L. J. and Ertel, N. H.—(1969) *Ann. intern. Med.*, **70**, 565.

Stewart, H. L., Snell, K. C., Dunham, L. J. and Schlyen, S. M.—(1959) 'Transplantable and Transmissible Tumors of Animals'. Washington, D.C. (Armed Forces Inst. of Pathology).

Young, N. F., Kensler, C. S., Seki, L. and Homburger, F.—(1947) *Proc. Soc. exp. Biol. Med.*, **66**, 322.